# Supplementary material for: Visual social information use in collective foraging
Source: PLoS Comput Biol. 2024 May 3;20(5):e1012087. doi: 10.1371/journal.pcbi.1012087 (PMC11095736; doi:10.1371/journal.pcbi.1012087)
Supplement: S1 Text — (PDF) [file pcbi.1012087.s001.pdf]

S1 Text for:

Visual social information use in collective foraging

David Mezey[1,2,*], Dominik Deffner[2,3,*], Ralf HJM Kurvers[2,3], Pawel Romanczuk[1,2]

1 Institute for Theoretical Biology, Humboldt University Berlin, Berlin, Germany
2 Science of Intelligence Excellence Cluster, Technical University Berlin, Berlin, Germany
3 Center for Adaptive Rationality, Max Planck Institute for Human Development, Berlin, Germany

* mezeydavid@gmail.com * deffner@mpib-berlin.mpg.de

1 Effect of changing resource density

Throughout the baseline simulations (see Fig 2) a fixed number (N_R^{TOTAL}) of resource units was distributed in different number of patches. We studied how the patchiness (i.e. the number of resource patches N_R in which the resource units are distributed) influences optimal social information use. Changing the total number of resources in the environment influences our results by tuning how persistent resource patches are and by that, how profitable social cues are.

If more units are distributed in the same number of patches, agents need more time to exploit a patch to depletion. This is an equivalent problem to decreasing the exploitation rate of agents, i.e. how many resource units they can collect from the environment per time step. With more collectable resource units per patch (and the resulting longer exploitation times especially in smaller groups) social information becomes more valuable as (1) it is more persistent and therefore easier to follow and (2) once followed provides a larger/longer term benefit for joining agents. In contrast, if less resource units have to be distributed in the same amount of patches agents can deplete these faster. As a result, social information loses value especially in large groups, because (1) it becomes ephemeral and therefore difficult to follow and (2) even if successfully followed, provides less/shorter term benefits. This modification thus only changes the scale of the results presented in Fig 2 of the main text, i.e. it produces a quantitative difference in the value of social information, but leaves the reported qualitative pattern unchanged.

To show that the overall results are robust for different numbers of overall resources,we repeated our baseline simulations with half as many ($N_R^{TOTAL} = 1200$) and twice as many ($N_R^{TOTAL} = 4800$) total resource units as before for group sizes ranging from 5 to 50 agents.

We found that changing the total number of resource units to half or twice as many than what was previously presented in Fig 2 does not change the general trend of optimal visual social information use, that is, the patchier the environment agents have to navigate in, the higher the optimal social excitability parameter is (see Supporting Information S1 Fig).

In general, with more total distributed resource units (S1 Fig bottom row, $N_R^{TOTAL} = 4800$) high social excitability values (y axis, top) yield higher search efficiencies than with lower number of resources (S1 Fig first row) in the environment

for all group sizes used (S1 Fig columns) supporting our considerations about an increase in the value of social information in richer environments. As again, rich environments with many resource units allow groups to (1) rely on more persistent social information and (2) benefit from them longer term once followed. A particularly large difference can be observed for the patchiest environments ($N_R = 1, ..., 5$) where the optimal efficiency values were shifted towards higher social excitability parameters as N_R^{TOTAL} increased. Similarly, the other way around, with less resource units distributed in the environment (S1 Fig first row), less social strategies (y axis, bottom) generally achieved higher efficiency values than that in richer environments (S1 Fig second and third rows) especially in uniform environments (N_R=20,...,100) showing how social information lost value with (1) more ephemeral patches and social information and (2) less beneficial joining events coming from the poor quality of patches. Our results show that the richness of the environment indeed scales the (1) availability and (2) total benefit of social information in our framework, but the general trend we present in our manuscript holds for a wide range of environments in terms of richness. The effect of such manipulation of the environment is particularly impactful in edge cases. For instance, in case all patches contain 0.25 resource units (which is just enough for a single agent to collect in a single time step) social information will be fully devalued within the current framework. It will not be followable as from almost all distances, it is impossible to reach the patch within a single time step. Furthermore it will not hold any benefit for agents as even if arriving to the patch it will be surely depleted in the meanwhile. On the other side of the spectrum, with patches holding an infinite amount of resource units, at least with the current framework, agents will be "stuck" in an exploiting state as soon as they find a patch. In this case, on long timescales the collective return will be similar, irrespective of how social agents are.
