# Supplementary material for: Visual social information use in collective foraging
Source: PLoS Comput Biol. 2024 May 3;20(5):e1012087. doi: 10.1371/journal.pcbi.1012087 (PMC11095736; doi:10.1371/journal.pcbi.1012087)
Supplement: S1 Fig — Collective search efficiency normalized per environment/column (i.e. relative search efficiency) for different amount of total distributed resource units (NRTOTAL, rows), group sizes (NA, columns), social excitability (ϵw, y axis) and environments (Number of Patches, NR, x axis). Agents are more social with higher social excitability. The environment is patchier with less and more uniform with more patches. (PDF) [file pcbi.1012087.s002.pdf]

S1 Fig for:

Visual social information use in collective foraging

David Mezey[1,2,*], Dominik Deffner[2,3,*], Ralf HJM Kurvers[2,3], Pawel Romanczuk[1,2]

1 Institute for Theoretical Biology, Humboldt University Berlin, Berlin, Germany
2 Science of Intelligence Excellence Cluster, Technical University Berlin, Berlin, Germany
3 Center for Adaptive Rationality, Max Planck Institute for Human Development, Berlin, Germany

* mezeydavid@gmail.com * deffner@mpib-berlin.mpg.de

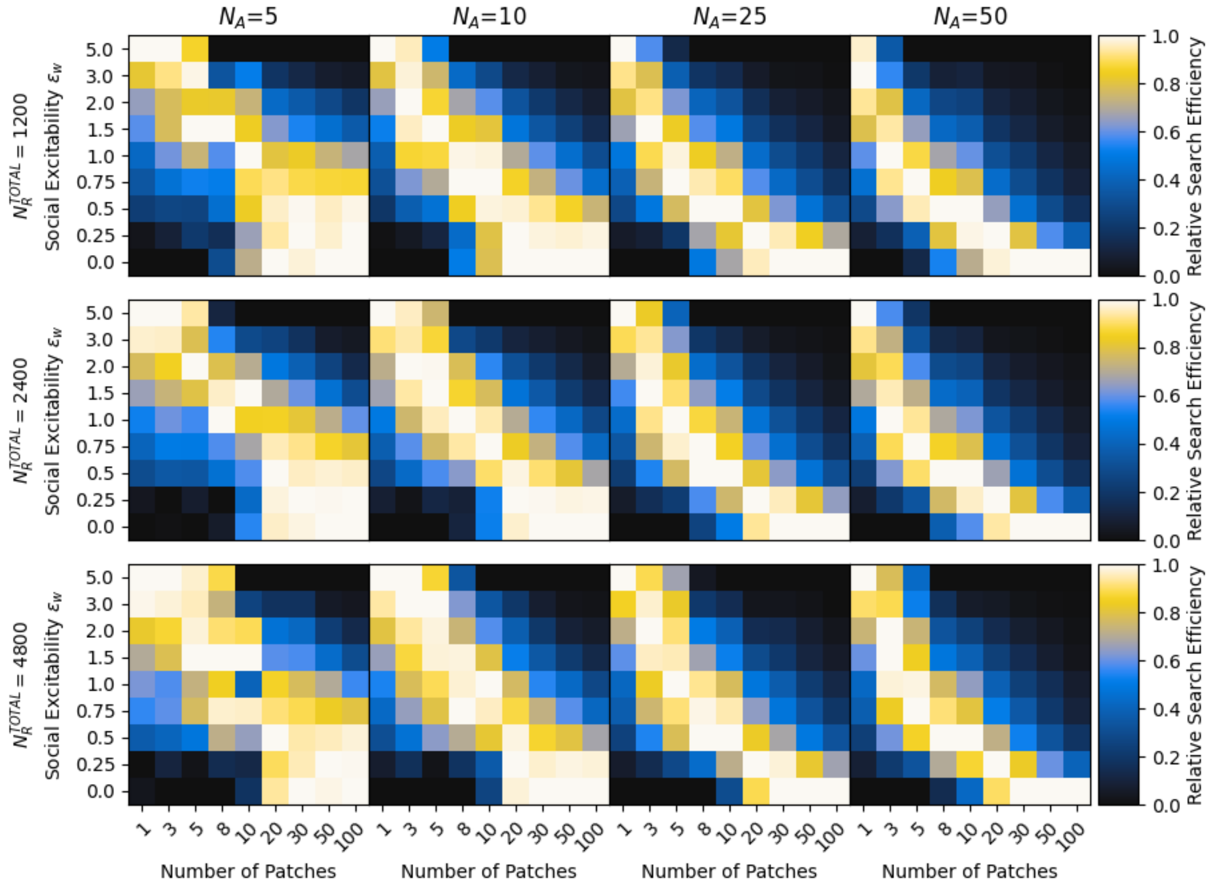

Fig 1. Effect of changing resource density Collective search efficiency normalized per environment/column (i.e. relative search efficiency) for different amount of total distributed resource units (N_R^{TOTAL}, rows), group sizes (N_A, columns), social excitability (ϵ_w, y axis) and environments (Number of Patches, N_R, x axis). Agents are more social with higher social excitability. The environment is patchier with less and more uniform with more patches.
