# Supplementary material for: Visual social information use in collective foraging
Source: PLoS Comput Biol. 2024 May 3;20(5):e1012087. doi: 10.1371/journal.pcbi.1012087 (PMC11095736; doi:10.1371/journal.pcbi.1012087)
Supplement: Attachment — Submitted filename: ResponseLetterMezeyetalPLOSCB.pdf [file pcbi.1012087.s005.pdf]

Dear Dr. Ricardo Martinez-Garcia and Dr. Zhaolei Zhang,

Thank you for the opportunity to resubmit our manuscript, "*Visual social information use in collective foraging*" to PLOS Computational Biology and for the constructive feedback of the editorial board and the reviewers. Your reviews allowed us to improve the quality and impact of our work in many aspects. In response to the comments and suggestions, we have carried out a thorough revision of our manuscript.

Most importantly, to accommodate Reviewer 3's important observations, we expanded our discussion to describe how our model differs from simpler phenomenological ones, how our results are connected to selective information use and that, in future research, one could investigate the effect of other types of constraints not studied in this work.

Following the editors' guidance and suggestions of Reviewer 2, we have conducted additional sensitivity analyses testing the robustness of our results for different resource densities (i.e. the total number of resource units in the environment). The results show that changing the total number of resource units modifies the overall value of social information across all environments, but leaves all the key results qualitatively unchanged.

We also addressed all other comments made by the four reviewers (see detailed revision summary below). This included clarifying the mathematical formulations of our model, refining some of our definitions, and improving our figures' readability.

We think that these revisions have significantly strengthened our manuscript, and we are looking forward to its possible publication in PLOS Computational Biology.

Best Regards,
David Mezey
(on behalf of all co-authors)

Detailed Revision Summary

Reviewer #1:

In their manuscript ”Visual social information use in collective foraging”, the authors simulate an agent-based model of social particles in search for patches of resources, with an increasing level of complexity. Each searcher can be in either an exploration state, a social relocation state or an exploitation state, and it switches between these states based on the information it collects along its trajectory, both from its own exploration and from the observation of other agents' behavior. The authors first quantify the search efficiency as a function of the sensitivity to social stimuli in an idealized setting with optimal vision, and then refine their findings by studying the effect of visual occlusion, restricted field of view and collisions with other agents. They find that these latter effects modify substantially the results in the idealized case and argue that optimal strategies, and in particular the optimal social sensitivity, depend greatly on such physical constraints are a trade-off between various phenomena.

Overall, the manuscript is very interesting, well written and perfectly relevant for the current state of the art. The warning the authors make on the fragility of optimal search strategies derived in idealized scenarios when one adds realistic constraints is particularly significant.

R1.1:

Thank you very much for your insightful review and for recognizing the importance of our work. We deeply appreciate your comments on the manuscript's relevance to the current state of the art and to our work’s significance regarding the fragility of model predictions derived from idealized scenarios.

However, my main negative comment on the manuscript is that most of the quantities that are used are not defined properly in the main text. I understand that the details are reported in the supplemental material, but it is very frustrating to have absolutely no quantitative definition of the model in the main text. This gives an impression of vagueness for the reader and I strongly believe that some mathematical details should be added in section 1.

R1.2:

Thank you for your constructive and detailed feedback regarding the placement and clarity of the mathematical formulation of our model. Our goal was to provide a more accessible, higher-level overview of the model in the initial sections of the manuscript (before the main results) and move the detailed mathematical formulations to the Methods section in the end of the main text (not the Supplementary Material). By that we tried to make the paper more

approachable for readers not primarily focusing on the modeling details, while still offering comprehensive details later for those with a deeper interest in this subject.

Taking your feedback into account, we have made substantial revisions to our manuscript. We have now incorporated key quantitative definitions and essential mathematical details in Section 1. This adjustment ensures that readers receive a clear understanding of our model's framework early on, enhancing the manuscript's coherence and addressing the concerns of vagueness, while maintaining accessibility for a diverse audience.

Here are the main ones missing, to my opinion:

We made the following changes to address your comments:

- In the exploration state, the agent is said to perform a "random walk", but it should be a bit more explicit.

R1.3:

We now clarify this explicitly in the results section as follows (line 105-108):

> "*In the exploration state agents move according to a random walk* ***choosing their change of heading angle from a uniform distribution between ±θ_expmax with θ_expmax=0.5 rad in each time step****, while collecting personal and social information.*"

- The authors mention 25000 time steps in their simulations, but what is a time step here?

R1.4:

Here, as we did not model any specific experiments, the time steps (ts) as well as spatial displacement (px) are given in arbitrary units. If a specific real-world system is to be modeled, they can be chosen to represent any time step and distance in SI units. For example, one could identify one time step with 1s and one pixel with 1dm (decimeter). In this case, the currently set speed of the agents would correspond to 3dm/s=0.3m/s.
In our simulation framework, a time step is the basic unit of time, i.e. a single iteration in which (1) all agents collect the available environmental information (social and private), (2) update their evidence accumulation processes, (3) make a decision of the 3 available states accordingly and (4) if possible according to the simulation parameters and the state of the agent also interact with the environment (movements, exploitation actions, resolving collisions) as explained in details in the methods section. We now also explicitly clarify this in the results section as follows (line 147-150):

*"[...] **In each simulation time step agents collect and accumulate available information (social and private), update their behavioral state accordingly (exploration, relocation or exploitation) and interact with the environment if applicable (exploitation, movement, collision).** We used T = 25000 time steps per simulation run and repeated our experiments 10-80 times (with more repetitions for small groups and patchy environments to account for inherently higher stochasticity while limiting computing resources)."*

- How do u(t) and w(t) evolve ? We can see in figure 1)c) that w increases smoothly, but not u, why is that ? And how are their threshold values decided ?

R1.5:

The evidence accumulation processes, u(t) and w(t), collect and accumulate private and social information, respectively. Social information for the focal agent is defined as the mean of its social visual projection field at every timestep, i.e., a value between 0 and 1 proportional to (1) the number of exploiting agents visible and (2) their proximity to the focal agent. Consequently, social information is accumulated smoothly/gradually by nature. On the other hand, private information comprises two components: (1) a large initial component for newly discovered patches that fades away on short time-scale (Tn=10 time steps), and (2) for patches that are not newly discovered, a smaller, long-term component corresponding to the resource units collected per time step from the patch until the agent leaves the patch or the patch is fully depleted (in our simulations, these two are synonymous). For the mathematical formulation of private information, please refer to Section 'Private Information' in the Methods.

The difference in the initial rise of w(t) and u(t), upon encountering social or private cues, stems from this key distinction between how we define social versus private information. Specifically, it originates from the novelty component of private information, a feature absent in social information, which provides an initial 'boost' to the private integrator that fades within a few time steps.

We now clarify our design in the methods section as follows (line 668-679):

"The reason behind including a large, short-term, initial novelty component in the private information is to allow agents to start exploiting a newly discovered resource patch upon finding it. In case the novelty component was not added to the personal information, it would only consist of the collected resource units per time step giving rise to a vicious circle: Agent's wouldn't receive personal information until starting exploiting a patch, but only incoming personal information, i.e. collected resource units would allow agents to start exploitation. Including the novelty component allows agents to initially "pool" the quality of the patch after which they can still choose to stop exploiting it depending on its quality. Throughout this paper, we did not experiment with resource patch quality and

> *kept it high enough for agents to keep exploiting patches (keeping u(t) above threshold) until depletion even after the initial large novelty component fading away.”*

For further clarity, we now emphasize this difference in the Results section and in the caption of Figure 1. as follows:

> *“Results (line 95-98): [...] Agents switch between these states based on the accumulation of personal and social Information over time (see Sec. Evidence accumulation and decision making). ~~Personal information includes the novelty of newly discovered patches and the amount of resource units consumed (See Sec. Private Information)~~.* ***Personal information consists of (1) a large initial novelty component making agents immediately start exploiting the patch upon new discovery, and (2) the amount of resource units consumed per time step (See Sec. Personal Information).”***

> *“Figure 1. Caption: [...] (A) An exploiting agent is visible and the individual’s social integrator w(t) increases* ***proportionally to the successful agent’s projection on the focal agent’s retina (i.e. to vicinity)*** *until reaching its threshold (grey dotted line). The focal agent then switches to social relocation (pink) and approaches the social cue (B). When reaching the patch, the agent’s individual integrator u(t)* ***receives a large novelty component from the newly discovered patch*** *and crosses its threshold. The focal agent starts exploiting the found patch* ***immediately****. Agents* ***keep receiving private information proportional to the patch quality*** *and they exploit the patch (C) until depleted”*

Choosing the threshold values for u(t) and w(t) is an equivalent problem to changing the weights accumulating personal or social information, i.e. ϵu and ϵw respectively. As an example, by lowering the threshold value for w(t) for the same amount of social information per time step and same social excitability ϵw, the process will reach the threshold earlier than with higher thresholds. Alternatively if we keep the threshold high but now increase ϵw, the incoming social information will be scaled up, hence, as a same result, the process will reach the now higher threshold earlier.

In our work, we fixed ϵu and other exploitation related parameters (such as the value of the novelty component or the resource patch quality) in a way that agents consistently exploit patches immediately upon finding them until fully depleting them. Although it is an interesting future direction to also experiment with such parameters biasing agents’ preference of richer patches over lower quality ones, we first wanted to use a simple exploitation behavior to be able to study social information use.
Regarding the social integrator, throughout the paper we systematically explored the effect of ϵw on the social integration process and the resulting group behavior.

We now clarify this in the section about evidence accumulation as follows(line 596-602):

> *"After accumulating individual and social information and updating both integrators accordingly, agents switch to the behavioral state that corresponds to their current neuronal activity (ui(t, xi , yi), wi(t)) based on thresholding rules (Equations 6-8). We define a positive threshold operator tr+ x giving 1 if decision process x is above it's decision threshold Tx, 0 otherwise. Similarly tr− x is only 1 if the process x is below its threshold.* ***We fixed Tu to 0.5 and other exploitation related parameters in a way that agents consistently exploit patches immediately upon finding them until fully depleting them. To explore the effect of varying strength of social information use we fixed Tw to 0.5 and varied ϵw systematically. Note that the exact decision of Tw does not influence the results qualitatively as it has the same effect as ϵw: it influences how fast (given a constant rate of incoming social information) w(t) reaches its threshold. Therefore, it's sufficient to fix one of them, and vary the other.***"

- How is \epsilon_w defined? This is a crucial quantity in the entire manuscript and there is no clear definition of it in the main text. Again, I understand that it is in the supplemental material, but there should be more details on it in the main text.

R1.6:

We agree with the reviewer that we did not define ϵw clearly in the results section, which might have led to difficulties in fully understanding our findings. We have now included the definition of social excitability ϵw in the revised text as follows (line 119-122):

> *"Importantly, a single parameter social excitability ϵw controls how sensitive agents are to social (versus personal) information.* ***This parameter serves as a weight scaling the incoming social information for the social integrator w(t) and, therefore, determining how quickly the visual social information is accumulated.*** *As a result, social excitability determines how socially agents behave (see Fig. 1d.)."*

- How is the search efficiency defined ? Is it related to the number of patches exploited by the end of the simulation ? Or is it a typical timescale ? The definition of search efficiency is an important question in all papers on search/foraging, it should be clearer here.

R1.7:

Thank you for highlighting the need for a clearer definition of search efficiency and for distinguishing between relative and absolute search efficiency. In response, we have now

explicitly defined the absolute collective search efficiency metric, E, in the results section as follows (line 153-155):

> *"We then quantified key metrics defined in Sec. Global Metrics with the primary metric being the absolute collective search efficiency of the group E, i.e., the average collected resource units per simulation time step and agent."*

Then we clarify the definition of relative search efficiency in the text and provide justification for such additional normalization per resource environment (line 159-166):

> *"Figure 2 shows the ~~relative~~ **collective** search efficiency (top row; see Eq.13 and Sec. Global Metrics), mean inter-individual distance (middle row; see Eq.14) and relative time spent in social relocation state (bottom; see Eq.15) for different group sizes (columns), values of the social excitability parameter ϵw (y-axis) and different environments (x-axis). **Search efficiency values are further normalized per environment (per column) to better present optimal values in environments, resulting in relative search efficiency values per environment between 0 (least efficient) and 1 (most efficient).** Brighter colors indicate higher values."*

Then we also include the clarified definition in the figure caption as follows:

> "Collective search efficiency **E normalized by columns** (**i.e. relative search efficiency**, ~~E,~~ first row), mean[...]"

To address this problem, I suggest that the authors add a section before the Results section where they properly introduce the model. This would help a lot the reading experience and allow the reader to understand more profoundly the results shown later in the manuscript. I also add other comments and questions.

R1.8:

As noted earlier, we prefer to split our model description to a higher-level, less detailed overview and a detailed methods section. To clarify the structure for the reader, we now start the results section by immediately referring to the methods section for a detailed mathematical description while clarifying that the overview in the results section is less detailed (line 81-84).

> *"To study visual social information use in collective foraging we created a mathematical model combining spatially explicit movement and internal evidence accumulation. For a detailed description see Sec. Methods and Materials. In the upcoming section we provide a high-level overview. In our model, groups of disk-shaped agents[…]"*

- What is the size of the simulation box with respect to the size of the agents and/or of their visual perception radius ? Could the authors express the number of agents and patches in terms of their density or packing fraction ?

R1.9

We appreciate the reviewer's suggestion to present our parameters in terms of density and packing fraction, providing a clearer understanding of the simulation environment relative to the agent and patch sizes. We now add an example of what proportion of the arena is covered by agents and patches (line 140-146):

> *"Agents' radius was fixed to RA = 10 pixels in a square arena with border length of W = H = 500 pixels yielding a single agent covering ca. 0.126% of the arena. The resulting packing fractions (i.e. the proportion of arena surface covered by agents without any overlap) range from ca. 0.004 for NA = 3 to 0.126 for NA = 100 agents. Resource patches had a fixed size (unless explicitly stated differently) with radius RR = 15 pixels yielding packing fractions from ca. 0.008 for NR = 3 to 0.28 for NR = 100 patches."*

Throughout this paper agents' visual range was fixed to be larger than the arena diagonal. In other words, agents throughout all experiments had an unlimited visual perception radius. We now clarify this in the main text as follows (line 116-117):

> *"If agents observe successful others within their visual field and the social integrator w(t) reaches its threshold, agents switch to the social relocation state and move towards visible exploiting agents (See Sec. Movement; shown in pink in Fig. 1).* ***Agents have an infinite visual perception radius; that is, they can "see" successful others regardless of the distance from them."***

- How does the rate of exploitation of resources impact the results ? Could the author authors discuss this, at least qualitatively?

R1.10

The rate of exploitation was consistently set at 0.25RU per timestep across all experiments. We agree that varying this parameter might influence our results in two ways:

(1) With a higher exploitation rate agents in our framework would receive higher amounts of personal information during exploiting patches. In case the weight of personal integrator ϵu wouldn't have been fixed throughout the paper or we had a non-zero cross-inhibition between u(t) and w(t) this would introduce a larger bias towards personal information use. Note that this can be achieved by changing e.g. the threshold of the personal integrator, its integration weight ϵu or the cross inhibition from integrator u(t) to w(t), i.e.

S_uw. In our initial investigations, on the other hand, we did not consider such modifications, simply because they result in more complex behaviors (such as patch leaving) and they are out of the scope of the present paper in which we specifically focused on optimal social information use.

(2) A higher rate of exploitation also influences how persistent resource patches are, and by that how often new patches are regenerated. A higher exploitation rate reduces the lifetime of the patches. With shorter patch lifetimes social information (i.e. visible exploiting agents) becomes scarcer and more difficult to follow. The same effect would be caused by a lower total number of distributed resource units in the environment, as it would cause patches to contain less resource units and by that they would be faster depleted. We have addressed this crucial aspect, previously omitted from our discussion, in response to Reviewer #2 and included the analysis results in the supplementary material for comprehensive insight (See section R2.1).

Reviewer #2:

The paper is well written. The results are clearly described, specifically the demonstrative simulation frames of different scenarios provide great insights. Methods are properly documented.

Thank you very much for your thoughtful review. We are particularly pleased that you find our demonstrative simulation frames informative.

My main remark is that across all simulations, the total number of resource units in the environment remains constant. While the authors varied between “patchy” and “uniform” environments, it would be equally interesting to see the results for “dense”(high) and “sparse” (low) environments (based on the total number of resources).

R2.1

Changing the total number of resources in the environment might indeed influence our results by tuning how persistent resource patches are and by that, how profitable social cues are. To summarize, if more units are distributed in the same number of patches, agents need more time to exploit a patch to depletion. Note that this is an equivalent problem to decreasing the exploitation rate of agents, i.e. how many resource units they can collect from the environment per timestep (See section R1.10). With more collectable resource units per patch (and the resulting longer exploitation times especially in smaller groups) social information becomes more valuable as (1) it is more persistent and therefore easier to follow and (2) once followed provides a larger/longer term benefit for joining agents.

In contrast, if less resource units have to be distributed in the same amount of patches agents can deplete these faster. As a result, social information loses value especially in large groups, because (1) it becomes ephemeral and therefore difficult to follow and (2) even if successfully followed, provides less/shorter term benefits. This modification thus only changes the scale of the results presented in Figure 2., i.e. it produces a quantitative difference in the value of social information, but leaves the reported qualitative pattern unchanged.
To show that the overall results are robust for different numbers of overall resources,we repeated our baseline simulations with half as many (NRTotal=1200) and twice as many (NRTotal=4800) total resource units as before for group sizes ranging from 5 to 50 agents. We added our results as a separate supplementary material page (*see Sup. Mat. 1. and Sup. Fig. 1*) and we also refer to our extended analysis in the main text as follows (line 182-185):

> *“Varying the total number of available resources units (NRTotal) changed the overall value of social information across environments, but left the qualitative results unchanged (see Supporting Information S1).”*

In relation to that, when exploring sparse environments, it is usually beneficial to implement an adaptive random walk : “Nauta, Johannes, et al. "Enhanced foraging in robot swarms using collective lévy walks." 24th European Conference on Artificial Intelligence (ECAI). Vol. 325. IOS, 2020.”
As relevant citations, the authors may include this paper, as it also highlights how physical constraints (avoiding collisions) on the individual-level enhances the collective foraging performance.

R2.2

We are grateful to the reviewer for highlighting the importance of this piece of literature that we simply missed during our literature search. Due to its high relevance we included the paper in the results section about colliding agents as follows (line 332-339):

> *“As a result, maladaptive “traffic-jam” scenarios around exploiting agents occur less often, and collectives maintain efficient parallel exploration of the environment.* ***The dynamic of physical constraints indirectly facilitating more efficient group behavior aligns with observations previously made for foraging robots[34]. Engineering collective Levy walks to avoid collisions at the individual level (instead of avoiding them due to visual occlusion) also enables swarms to significantly improve their foraging efficiency. Such strategies, utilizing physical constraints to enhance group performance, show the crucial role these constraints might play in optimizing collective behavior.”***

Results 1.1 (Fig 2)

I would prefer if the x-axis shows the patch density instead of the number of patches, since this is related to the environment size.

R2.3

Although we prefer to keep the number of patches as the x-axis (as this straightforwardly describes the resource landscape with a fixed total number of resources), we recognize the importance of clarifying the relationship between patch number and density given a constant arena size. We provide clarifications accordingly detailed in section R1.9. We also clarify better how we fix the total number of resource units. For further details see section R2.4.

It is unclear to me where the "transition" from a "patchy" to a "uniform" environment occurs. If this solely happens by increasing the number of patches, then this means the total amount of resources is constant throughout these experiments? I see this is stated in the Methods but while reading the Results this is confusing to me. I prefer a different variable than "number of patches" to make the transition clear.

R2.4

Throughout the paper the total number of resources (NR_Total) was constant. By that, changing the number of patches in which resource units are distributed will change the "patchiness" of the environment. We previously tried to provide sufficient information regarding how we changed the resource landscape in the main text as follows:

> *"[...] we systematically varied the social excitability parameter of agents and investigated its effects across different environments. In relatively "patchy" environments, resource units were concentrated in few but rich patches. In relatively "uniform" environments, the same number of units was distributed across many but poor patches."*

We now see that drawing a clear distinction between patchy and uniform environments might cause confusion if it's not described in more detail how we used the same amount of resource units and how we changed the environment smoothly between relatively patchy and uniform environments. The revised version is as follows (line 131-135):

> *"[...] we systematically varied the social excitability parameter of agents and investigated its effects across different environments* ***from patchy to uniform resource distributions****.* ***While keeping the total number of resource units constant, we varied the environments by systematically changing the number of resource patches in which they were distributed between N_R=3 to N_R=100 patches.*** *In relatively "patchy" environments, resource units were concentrated in few but rich patches. In relatively "uniform" environments, the same number of units was distributed across many but poor patches."*

Regarding the selected environments for our figures 3, 4 and 5, we use the name "Patchy Environment" for environments with N_R=3 patches, "Intermediate Environment" for N_R=8 patches and "Uniform Environment" for N_R=50 patches. This is clearly stated in each figure but might not have been clearly visible due to small labels fixed in the upcoming section R2.5.

Results 1.2 (Fig 3)
Confusing labels of the columns like "Patch environment NA=3" while the row indicates NA=5

R2.5

We see how small labels could have been mistaken for NA for both the rows and columns of the figures. The rows show different group sizes where the number of agents (NA) was varied row by row. The columns show different environments where keeping the total number of resource units fixed the number of resource patches (NR) was varied column by column. We solved the issue with the visibility of small labels by (1) increasing the figure width (to full width as they were originally designed for this size), (2) removing figure margins where possible and (3) increasing all labels on all the figures. For a better comparison, please see the old and new version of Figure 3 below.

Previous version of Figure 3. with small labels:

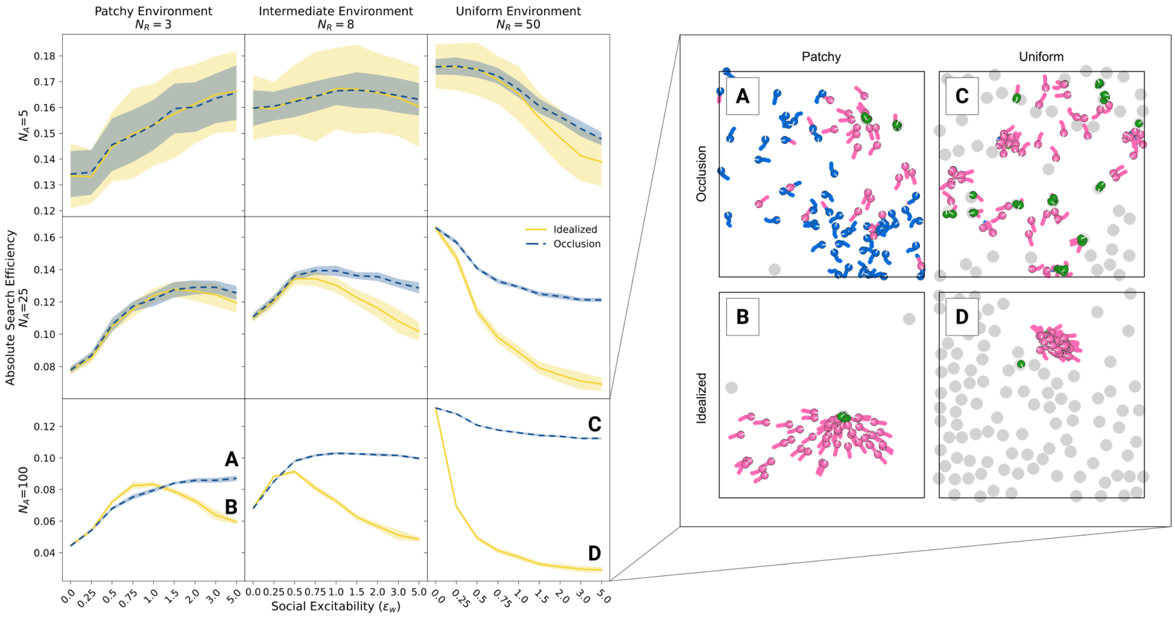

New version of Figure 3. with larger labels:

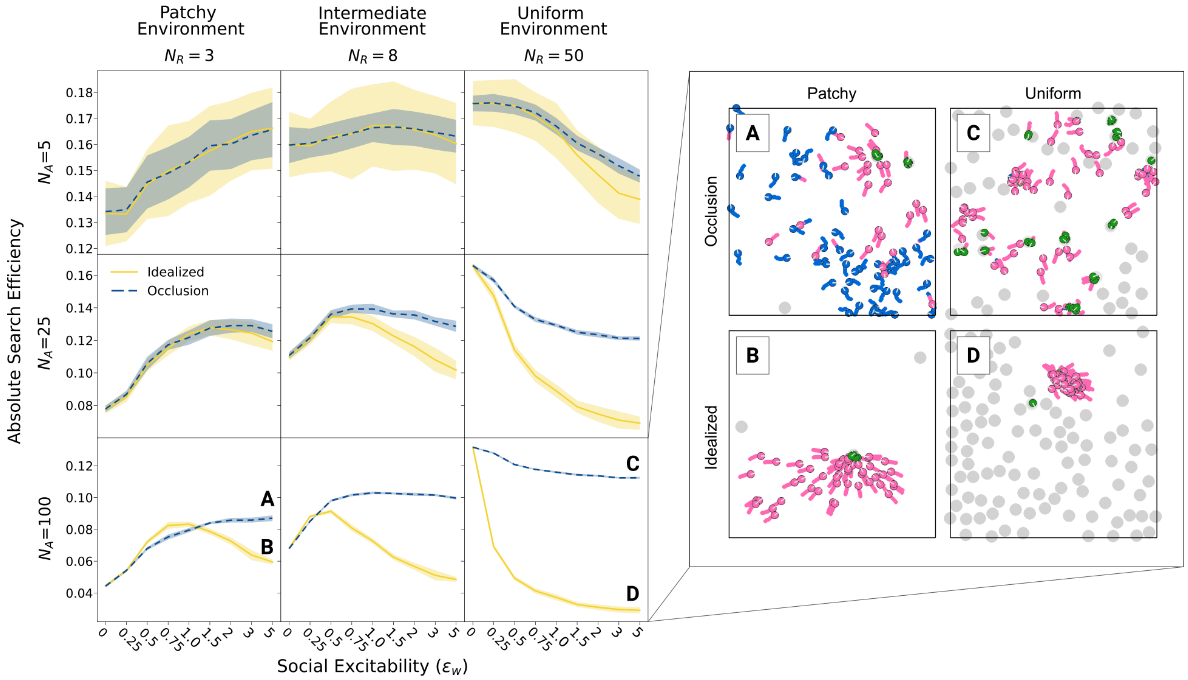

The “absolute search efficiency” is shown here, while in Fig 2 the “collective search efficiency” is measured (but the label shows “relative search efficiency”). What are the differences between these?

R2.6

Thank you for calling our attention to the missing definition of relative search efficiency and the corresponding mistake in our figure caption. We addressed this point by clarifying the definition of search efficiency in the main text and by adding a detailed description of how the relative search efficiency (normalized across conditions within the same environment) is different from the original definition. As this point was also raised by Reviewer #1, for the detailed revision please see our reply for Reviewer #1 in section R1.7.

How is the value of L decided? Is it inspired by certain animal parameters?

R2.7

In our framework L defines the half size of the visual field of view (FOV), in other words, agents FOV span from -L to L. To uncover the effects of a limited field of view, we systematically changed L between 0 and pi, resulting in agents FOV spanning between 0 and 2pi. The selection of L values was not directly inspired by specific animal parameters but chosen to broadly explore the theoretical space of FOV limitations. For our results summarized in Figure 4. we used 20 equidistant values for L from 0 to pi to cover the whole spectrum of possible FOVs.

In general, the labels in some Figures are relatively small and hard to read

R2.8

We thank the reviewer for pointing out the issue with the readability of labels in some figures. We fixed this by increasing the labels and the figure size. For the detailed revision please see section R2.5.

Reviewer #3

This paper builds a social foraging model based on previous phenomenological models, and adds a mechanistic component to them, which is largely related to the constraints of the physical world, like occlusion, limited vision field, and collision.
This paper is a good contribution to foraging models, especially due to the underlying evidence accumulation mechanism and formalizing physical constraints that will be valid for many (not all)

collective foraging systems. Some comments below for the authors to clarify and to possibly add to their paper:

Thank you very much for your thorough and helpful review. We were delighted to read your insights especially on selective information use. We hope we could accommodate all your points with our revision.

1. Does occlusion work to limit social information in a similar way as any general metric of distance?

R3.1

In our framework distance from the social cue (i.e. from an exploiting agent) influences how large the social cue appears for the focal agent (closer exploiting agents being larger). As a consequence, distance from exploiting agents influences the amount of incoming social information and by that it determines how fast (if at all) the focal agent will follow a social cue. In other words, we define the social information as the mean of the focal agents' social visual projection field. If an exploiting agent is close to a focal one, its corresponding visual projection (See Fig.1.a.) will be wide, resulting in a large amount of incoming social information, biasing agents towards the social relocation state more. If an exploiting agent is far from a focal one, its corresponding visual projection will be narrow resulting in less incoming social information, and in later or, depending on the social excitability parameter, no social relocation at all. Social information, being visual, therefore scales with the number of visible exploiting agents and the distance from them according to the laws of optics. For further information on how social and personal information is defined please also see section R1.5 and our updated Results and Methods section.

Occlusion has a similar but not the same effect. On one hand visual occlusion has a distance-dependent effect: If the distance is large between the focal agent and a social cue, there is simply higher chance that (1) there will be an exploring agent between these and (2) this third agent occludes social information from the focal one being the social cue a narrow projection (given that it's far away from the focal one). On the other hand, visual occlusion also has a large effect in shorter distances. For instance, it can happen that a focal agent can not take even a nearby social cue into account because another agent between the focal and the exploiting one occludes this visual information. Note, that only based on distance the focal agent would receive large amounts of social information but due to the visual occlusion this is not possible anymore. There's a higher likelihood of such occurrences in scenarios where the group is densely packed in a small area. Hence, beyond distance-dependent effects, visual occlusion introduces a change in the availability of social information based on (local) group density, rather than solely on distance. For a quantitative demonstration, please see Figure 3, where the difference between the idealized (yellow) and more realistic case with visual occlusion (blue) widens as group size increases (rows). Since we maintain a fixed arena size, this equates to

increasing group densities. Furthermore, this difference escalates with higher social excitability (x-axis), causing agents to cluster around resource patches and locally heighten group density.

How will the results be different if agents were less likely to follow a social cue based on the energetic cost (based on distance) of following the cue?

R3.2

Thank you for your question. This is already implicitly taken into consideration according to how the social information is scaled with distance (see section R3.1). With the same social excitability parameter agents only follow a social cue if it is close enough (if it creates a large enough visual projection) to push the social integrator above its threshold.

Furthermore, once agents are in a social relocation state they steer towards social cues according to a simple maximum steering mechanism. In a nutshell this guides agents towards the direction (left or right) that has the most social information, always giving preference to nearby exploiting agents over farther ones.

It can hypothesized that even without occlusion, many organisms will not follow a cue very far away, because of a very real, very physical-world constraint of energy. So, what are some different real-world constraints which can lead to effects like the ones modeled here? I emphasize this point more in my last comment.

R3.3

We share the opinion of the reviewer that the combination of different environmental, perceptive or cognitive constraints will fundamentally change model results. We tried to emphasize this explicitly as one of the main findings of our paper in the discussion as well.Consequently, we agree that introducing energetic constraints could indeed influence how agents make decisions and navigate in the environment, with or without visual occlusions. Besides energetic constraints, we would also find it interesting in the future to investigate the effects of distance-dependent visual (or other perceptive) noise, individual cognitive biases and movement constraints such as more complex individual search strategies. Our current study primarily focused on fundamental visual constraints such as visual occlusion and a limited FOV. We now shortly describe this interesting line of future research in the discussion as follows (line 427-431):

> *"In the future, researchers might use controlled experiments as well as fine-grained tracking data to investigate how behavior generated by different evidence accumulation parameters maps onto optimal foraging predictions in naturalistic collectives with different perceptual abilities and movement patterns.* ***Furthermore, future studies might investigate fundamentally different constraints such as energetic costs***

> ***limiting the behavior of most living and engineered agents or constraints of other sensory modalities such as olfaction or audition."***

2. Line 109 – what is the rationale behind manipulating group size and resource distributions? Is it simply based on the previous models cited here, or is there something more specific based on the physical constraints?

R3.4

We manipulated resource distribution as this serves as one of the main drivers of social information use in collective foraging and it is widely studied in the literature. Our aim was to demonstrate that our results are robust across different group sizes, thus we systematically varied the number of agents in our experiments. Additionally, changing group size helped us explore how certain realistic constraints (such as visual occlusion, limited field of view, or collisions) are affected by group density. Both visual occlusions and collisions become more prevalent in larger groups within the same-sized arena, amplifying their effects as group size increases, as detailed in the results section (line 236):

> *"If groups are small, there are few other agents in the environment that might block the agents' FOV. In this case, simulations including occlusion largely mirror the more idealized scenario: high social excitability is beneficial in patchy environment and low social excitability is beneficial in uniform environment. As groups become larger and there are more agents potentially blocking others' vision, the role of occlusion becomes evident."*

For a detailed description of group size-dependence of the effect of visual occlusions, see section R3.3.

3. Line 119 – why was T= 25000 used? Were there any time-dependent effects, which then went away over T?

R3.5

The duration of each simulation run (T=25000) was chosen to ensure that any initial effects dissipate across all group sizes and environments, given the parameters specified in Table 1. These parameters determine the basic dynamics of the simulations, such as agents' movement speed, the rate at which a patch can be exploited by one or many agents, etc. We tailored the length of the simulations taking these parameters into account, allowing the system to fully unfold (no qualitative change anymore) with all environments and groups sizes and to provide enough data for our analysis presented in the Results section.

4. Line 155 – "in small groups.. social information tends to be sparse. As a consequence.. high levels of social excitability is beneficial" is this also because social information is also spread out and slow to amplify in small groups? More on this in the next point

R3.6

We state the following (line 194):

> *"Second, in small groups, where other agents only rarely discover new patches, social information tends to be comparatively sparse. As a consequence, a high level of social excitability is required to make agents switch to social relocation and provide the benefits of adaptive social information."*.

This approach focuses on the fewer individuals in small groups who can serve as social cues once they find a patch. The distance from successful others (and by that the spread of social information) depends not only on group size, but also on how social agents are and which environment they navigate in. If agents are more social they tend to quickly move towards successful group members decreasing interindividual distances on average. Otherwise they explore the environment alone according to the random search process resulting in groups more spread out. Alternatively, instead of the number of agents, one could consider how the number of patches influence the dilution of social cues. If there are less patches there is a higher chance that these are getting generated farther from each other than in cases where patches are covering most of the arena. In this case, indeed, social information will be more spread out because only those agents count as social information that are exploiting one of the few patches. In this case a higher social excitability can counterbalance social cues that are generally farther. We answer your point regarding amplification in section R3.8.

5. Line 216-220 – Authors say that visual occlusion by successful agents prevents group members from seeing the social information, which limits social information use and increases collective efficiency.

R3.7

We think that there might have been a misunderstanding regarding this point. We state the following (line 249):

> *"To better understand how this process of adaptive self-organization unfolds, imagine an agent that found a resource patch in a relatively uniform environment. In case of idealized vision, also agents far away perceive the social information in the environment and start relocating towards the discovered patch. The patch, however, will likely be depleted until others arrived and the collective will then be concentrated in a small part of*

> *the arena. This results in an inefficient exploration of the environment. With visual occlusion, in contrast, the first joiners (who are also more likely to arrive at the patch in time) will shield the social information from farther agents which keep exploring the environment (see also video 2. [30]).”*

In other words, we state that visual occlusion by ~~successful agents~~ **first joiners** prevent **farther** group members from seeing the social information, which limits social information use and increases collective efficiency. To clarify, the first joiners only occlude the social information until they are relocating towards the patch being exploited. As soon as they join others and switch to exploitation mode they too count as social cues for those agents (farther ones) that didn’t “see” the first successful group as they were visually occluded.

To avoid any misunderstandings regarding how occlusion affects the collective behavior of agents in our framework, we now further clarify this mechanism as follows (line 254-264):

> *“With visual occlusion, in contrast, the first joiners **relocating towards the patch** (who are also more likely to arrive at the patch in time as they are close) will shield the social information from farther agents which keep exploring the environment (see also video 2.[30]). **Once the first joiners reach the patch and start exploiting it, they also count as social information visible for now farther agents and the process starts again. This way agents join others layer by layer according to visibility, facilitating individualistic behavior for far away individuals while allowing social behavior for nearby ones.**”*

It is unclear to me how social information in the model does not lead to a cascading/amplifying effect. That is, if one agent (A) joins a successful agent (B) at a resource patch, won’t A also be now counted as a successful forager, and so on for whoever joins, as long as they find a resource?

R3.8

During most of our experiments agents could overlap on resource patches (except when we studied the effect of collisions). Due to the definition of the social visual projection field in our framework, two agents fully overlapping while exploiting the same patch produce the same amount of social information as a single visible exploiting agent would do. As a result, although social amplification is present (as agents usually don’t fully overlap), it is small in most cases due to simple, purely vision-based information acquisition. This only changes in simulations where collision avoidance was enforced on agents. In this case more agents joining the patch will produce more social information as they can not overlap anymore (especially in large patches where many agents fit without overlapping). Note, on the other hand, that the social information being visual, the angle from which the exploiting agents are observed influences

such amplification effects. It might happen, for instance, that from one angle, aligning agents seem to overlap, while from the other they don't.

Maybe, occlusion here is not really preventing agents from seeing others due to a "shield" of previous joiners. Rather, it would be due to any agent in between any other agent [hence the larger effect for larger groups]. This effect can then delay the joining of other agents to agent B and by the time, an agent can "see" B, the cue would have been extinguished by the early joiners, like A. In this way, the effect of occlusion then boils down to distance-based effect. The closer an agent is to a social cue, the more likely it is to not be occluded by other searching agents, and vice-versa.

R3.9

Indeed, visual occlusion has a distance-based and a group density-based effect, please see our detailed answer in section R3.1. In general, social information is more visible for closer agents as there is less visual occlusion possible and in less dense groups/areas, as there are less agents that could occlude visual information. The mechanism we described in detail in section R3.7 is a result of both, i.e. of a locally (depending on distance) increasing group density and a general higher rate of occlusion for far away agents. As the patch is first discovered the closest agents for which this cue is visible will start to relocate towards it, locally increasing the group density and by that "shielding" the cue from farther agents. As soon as these relocating agents join the patch, they become visible for now farther agents that can relocate towards them increasing the group-density around the patch again. We think that the clarifications made in section R3.7 also address this point.

6. Relatedly, how does the model fit in with the often-observed natural process of "social facilitation", by which foragers join in based on others presence at a patch, rather than actually see the patch. The real cue can be occluded, as long as the joiners are not, which now become social information.

R3.10

Thank you for raising this important point about inferring social information. In this study social information was solely defined as visible individuals exploiting a patch and agents could not "see the patch" from a distance unless other agents were exploiting it. We agree with the reviewer, that more complex inference mechanisms are widely observed in humans and other animals, and they might influence how social information is used. For instance, inferring social information by observing visible relocating agents that follow social information not visible for the focal one, might influence optimal social information use. On the other hand, modeling such inference of social information is not straightforward (especially based purely on vision) and it might introduce additional complexities that, at least for our initial investigations, were simply out of scope.

7. Minor: Fig. 3 legend says “saves the foraging efficiency of highly social groups” – might want to rephrase to something like “prevents collective foraging efficiency of highly social groups from diminishing”, or “maintains high levels of efficiency even in highly social groups.”

R3.11

Thank you for calling our attention to a potentially problematic wording. We now changed the figure caption as requested.

8. I find it interesting in in patchy and intermediate environments, collision avoidance improves efficiency for individual foraging (epsilon =0), compared to idealized version (top row). This is an interesting result, is there an explanation for this effect? I think it is because under low sociality, collision avoidance acts as a gradient sensing mechanism which leads agents to move to areas where there are few others. Collision + occlusion condition probably localizes subgroups of agents which search in parallel. Collision can help find areas without other agents, while occlusion will force them to find these areas within small distance. So it is kind of a self-organizing subgrouping.

R3.12

Although we find the idea of a local gradient sensing mechanism through collision avoidance fascinating, Figure 5. shows the opposite relation. In intermediate and uniform environments (middle and right columns) individual foragers (epsilon=0, dark blue) reach higher efficiency in idealized cases (first row, between ca. 0.08-0.125 in intermediate environment and ca. 0.135-0.2 in uniform environment depending on patch size) than with collisions (second row, between 0.045-0.11 in intermediate environment and ca. 0.115-0.2 in a uniform environment depending on patch size). The misunderstanding might have come from the small labels on the figures that were difficult to read. To remedy this we increased the labels and the overall size of all our figures as described in section R2.5.

9. Discussion Line 311: is there a reason why individual exploration and exploitation was modeled using two competing integrators?

R3.13

We chose a pair of competing integrator processes, one dedicated to integrate social, the other to integrate personal information. They are competing, as they (1) are connected via cross-inhibitory weights and (2) they result in fundamentally different behaviors when firing (social relocation vs exploitation). If none of them cross their thresholds agents are in an indecisive state and explore the arena for personal and social information. If both of them are

above threshold (in the current framework) exploitation is preferred over social relocation. Dividing the evidence accumulation into a pair of integrators instead of combining them into a single two-sided accumulation process can be justified by the fact that social and personal information are fundamentally different (even coming from different sensory modalities, i.e. visual vs. local sensory), hence they might be also weighted and accumulated differently. Having cross-inhibition between these allows us in the future to (1) untangle the process of evidence accumulation from the process of cross-inhibition and as a result (2) to explore a rich pool of behaviors through systematically changing the inhibition between the integrators in both directions giving rise to e.g. patch leaving behavior or preference towards high quality patches in the presence of other exploiting agents. Ultimately, we are interested in the competition between private and social information as a continuation of this research presented. Note, that in the current study, most of these complexities were heavily simplified by, e.g. fixing all personal integrator related parameters or by fixing the cross inhibitory weights between the integrators to 0. In this sense, the resulting model in this study (with no cross inhibition) is an independent race type integrator pair with additional higher level logic to decide on the resulting movement behavior (i.e. preference to exploit patch when both of the integrators are "firing"). In sum, we chose this design as it allows us to study rich and complex behavior in the future and because it can be justified by the 2 main types of evidence accumulated from the environment.

10. The paper's big push is the role of "perceptual" constraints which are modeled as visual collusion, field of view. But perception extends beyond vision. In real-world animal foraging (or even robots I suppose), there are other senses at play, like smell and sound which pick up cues, often in a gradient-dependent manner. How do other perceptual constraints matter? I think to some extent the way limited perceptual field and occlusion are modeled here would hold true for every sensory system. So, it is possible rephrase the terms to just say general perception, rather than vision-focused.

R3.14

Thank you for your insight. We find it intriguing to think of sensory modalities other than vision in terms of perceptual constraints. Nonetheless, in this paper we particularly focused on visual perception being the primary sensory input for many species including humans and robots. The furthest we would go with generalizing our results to other sensory domains is generalizing to those that produce similar long-range topological mapping of the environment to what vision does, such as echolocation for e.g. bats or LiDar maps for robots. We find generalizing occlusion and limitations in the sensory field and range to olfactory or auditory inputs particularly challenging, nonetheless these modes have their own type of sensory limitations different in nature than that of the visual ones we present. We now emphasize oder sensory modalities as possible future directions of research (line 427-431):

> *"In the future, researchers might use controlled experiments as well as fine-grained tracking data to investigate how behavior generated by different evidence accumulation*

parameters maps onto optimal foraging predictions in naturalistic collectives with different perceptual abilities and movement patterns. ***Furthermore, future studies might investigate fundamentally different constraints such as energetic constraints limiting the behavior of most living and engineered agents or constraints of other sensory modalities such as olfaction or audition.""***

11. While the model systematically shows how physical and spatial constraints affect social information use and consequently collective foraging efficiency, I can't help but wonder how much the overall results differ from previous models (more simpler and phenomenological ones). To my understanding, the biggest result here is that physical constraints can work as a self-organized adaptive mechanism to limit overexploitation of social information – which is great. But it still points in the same direction as the results from previous, simpler models, i.e., selective use of social information is necessary for maintaining high collective search efficiency by preventing maladaptive herding. So, probably a bigger picture takeaway from this study, when compared against other previous models, is that selective use of social information is a fundamental process by which collectives manage the benefits and pitfalls of social information. But the way in which this fundamental process is brought about can differ across systems, based on the specific environmental, physical, social constraints that the system is embodied/situated within. In other words, constraints of vision and occlusion should be thought of as one of the "real-world constraints". So, inadvertently, these results solidify the general findings from phenomenological models. I was hoping to find discussion around this point in the Discussion section. It will make the findings stronger if authors address this point and discuss other real-life constraints that can be applicable to collective foraging models and how they compare to the constraints in this paper.

R3.15

Thank you for raising this point. We very much agree that selective information use is key to avoid over-exploitation shown before in simpler models. On the other hand, we would like to emphasize the point that while in previous models selective information use originated from the cognitive processes of agents, we showed that with the same simple cognitive mechanisms, preceptive or environmental constraints rather than cognitive ones can facilitate the same mechanism. To distinguish between these aspects it is necessary to have models accounting for perception, cognition and environmental factors separately in contrast to simpler phenomenological ones. We now include this in the discussion as follows (line 414-424):

"Physical and sensory constraints affect the performance of social groups by effectively modulating the influence of social information and preventing maladaptive herding. Thus, the effective weight of social information has two contributing factors: A cognitive one, where individuals internally assign different weights to social versus non-social cues, and an ecological one, determined by the ability of individuals to interact with the (social) environment. This ecological factor can only partially and indirectly be controlled by

*individuals through their behavior. **This distinction demonstrates that selective information use, a key mechanism to prevent overexploitation and avoid maladaptive herding previously shown with phenomenological models[18], can be also driven by environmental and perceptual constraints rather than solely by cognitive decision-making.** By "embodying" selective social information use within the perceptual system, such apparent constraints might partly remove the need for more sophisticated social learning strategies and increase the value of social information across a wider range of organisms. **Our results show that to fully understand how animals selectively use information, we need models that account for the interplay between perception, cognition, and the environment, offering insights beyond those provided by simpler phenomenological models.** In the future, researchers might use controlled experiments as well as fine-grained tracking [...]."*

Reviewer #4

The paper is well-written and fills an essential gap in the area. I just recommend a very careful style review since there are some redaction mistakes (e.g. lines 45-47 pg 2).

R4.1

Thank you for your feedback about the significance of our work and for your review. We fixed the redaction highlighted mistake as follows (line 48-52):

~~"This family of models—widely used in cognitive science and psychology to describe the temporal dynamics of decision-making see [24] for a review—proposes that agents continuously gather evidence for different decision alternatives at a particular rate, until the threshold of one alternative is reached and the agent makes a decision[25].~~
***This family of models, which is widely used in cognitive science and psychology to describe the temporal dynamics of decision-making[24], proposes that agents continuously gather evidence for different decision alternatives at a particular rate. This process continues until the threshold for one alternative is reached, leading the agent to make a decision[25] .**"*

We also carefully proofread our manuscript once again to avoid any additional mistakes of this kind.

Also, it would improve the reading if the authors homogenized the way they make citations (e.g. line 487 pg. 15).

R4.2

Thank you for your comment about a unified citation method. We now changed all citations according to the citation style of PLOS Computational Biology.
