# Supplementary material for: Visual social information use in collective foraging
Source: PLoS Comput Biol. 2024 May 3;20(5):e1012087. doi: 10.1371/journal.pcbi.1012087 (PMC11095736; doi:10.1371/journal.pcbi.1012087)
Supplement: Attachment — Submitted filename: review_PlosCompBio.docx [file pcbi.1012087.s004.docx]

This paper builds a social foraging model based on previous phenomenological models, and adds a mechanistic component to them, which is largely related to the constraints of the physical world, like occlusion, limited vision field, and collision.

This paper is a good contribution to foraging models, especially due to the underlying evidence accumulation mechanism and formalizing physical constraints that will be valid for many (not all) collective foraging systems. Some comments below for the authors to clarify and to possibly add to their paper:

1. Does occlusion work to limit social information in a similar way as any general metric of distance? How will the results be different if agents were less likely to follow a social cue based on the energetic cost (based on distance) of following the cue? It can hypothesized that even without occlusion, many organisms will not follow a cue very far away, because of a very real, very physical-world constraint of energy. So, what are some different real-world constraints which can lead to effects like the ones modeled here? I emphasize this point more in my last comment.
2. Line 109 – what is the rationale behind manipulating group size and resource distributions? Is it simply based on the previous models cited here, or is there something more specific based on the physical constraints?
3. Line 119 – why was T= 25000 used? Were there any time-dependent effects, which then went away over T?
4. Line 155 – “in small groups.. social information tends to be sparse. As a consequence.. high levels of social excitability is beneficial” is this also because social information is also spread out and slow to amplify in small groups? More on this in the next point
5. Line 216-220 – Authors say that visual occlusion by successful agents prevents group members from seeing the social information, which limits social information use and increases collective efficiency. It is unclear to me how social information in the model does not lead to a cascading/amplifying effect. That is, if one agent (A) joins a successful agent (B) at a resource patch, won’t A also be now counted as a successful forager, and so on for whoever joins, as long as they find a resource?

Maybe, occlusion here is not really preventing agents from seeing others due to a “shield” of previous joiners. Rather, it would be due to any agent in between any other agent [hence the larger effect for larger groups]. This effect can then delay the joining of other agents to agent B and by the time, an agent can “see” B, the cue would have been extinguished by the early joiners, like A.

In this way, the effect of occlusion then boils down to distance-based effect. The closer an agent is to a social cue, the more likely it is to not be occluded by other searching agents, and vice-versa.

6. Relatedly, how does the model fit in with the often-observed natural process of “social facilitation”, by which foragers join in based on others presence at a patch, rather than actually see the patch. The real cue can be occluded, as long as the joiners are not, which now become social information.
7. Minor: Fig. 3 legend says “saves the foraging efficiency of highly social groups” – might want to rephrase to something like “prevents collective foraging efficiency of highly social groups from diminishing”, or “maintains high levels of efficiency even in highly social groups.”
8. I find it interesting in in patchy and intermediate environments, collision avoidance improves efficiency for individual foraging (epsilon =0), compared to idealized version (top row). This is an interesting result, is there an explanation for this effect? I think it is because under low sociality, collision avoidance acts as a gradient sensing mechanism which leads agents to move to areas where there are few others. Collision + occlusion condition probably localizes subgroups of agents which search in parallel. Collision can help find areas without other agents, while occlusion will force them to find these areas within small distance. So it is kind of a self-organizing subgrouping.
9. Discussion Line 311: is there a reason why individual exploration and exploitation was modeled using two competing integrators?
10. The paper’s big push is the role of “perceptual” constraints which are modeled as visual collusion, field of view. But perception extends beyond vision. In real-world animal foraging (or even robots I suppose), there are other senses at play, like smell and sound which pick up cues, often in a gradient-dependent manner. How do other perceptual constraints matter? I think to some extent the way limited perceptual field and occlusion are modeled here would hold true for every sensory system. So, it is possible rephrase the terms to just say general perception, rather than vision-focused.
11. While the model systematically shows how physical and spatial constraints affect social information use and consequently collective foraging efficiency, I can’t help but wonder how much the overall results differ from previous models (more simpler and phenomenological ones). To my understanding, the biggest result here is that physical constraints can work as a self-organized adaptive mechanism to limit overexploitation of social information – which is great. But it still points in the same direction as the results from previous, simpler models, i.e., selective use of social information is necessary for maintaining high collective search efficiency by preventing maladaptive herding. So, probably a bigger picture takeaway from this study, when compared against other previous models, is that selective use of social information is a *fundamental process* by which collectives manage the benefits and pitfalls of social information. But the *way* in which this fundamental process is brought about can differ across systems, based on the specific environmental, physical, social constraints that the system is embodied/situated within. In other words, constraints of vision and occlusion should be thought as one of the “real-world constraints”. So, inadvertently, these results solidify the general findings from phenomenological models.

I was hoping to find discussion around this point in the Discussion section. It will make the findings stronger if authors address this point and discuss other real-life constraints that can be applicable to collective foraging models and how they compare to the constraints in this paper.
